# Supplementary material for: Anionic Pulmonary Surfactant Lipid Treatment Inhibits Rhinovirus A Infection of the Human Airway Epithelium
Source: Viruses. 2023 Mar 14;15(3):747. doi: 10.3390/v15030747 (PMC10055697; doi:10.3390/v15030747)
Supplement: Supplementary file 1 [file viruses-15-00747-s001.zip › viruses-2188315-supplementary figures.pdf]

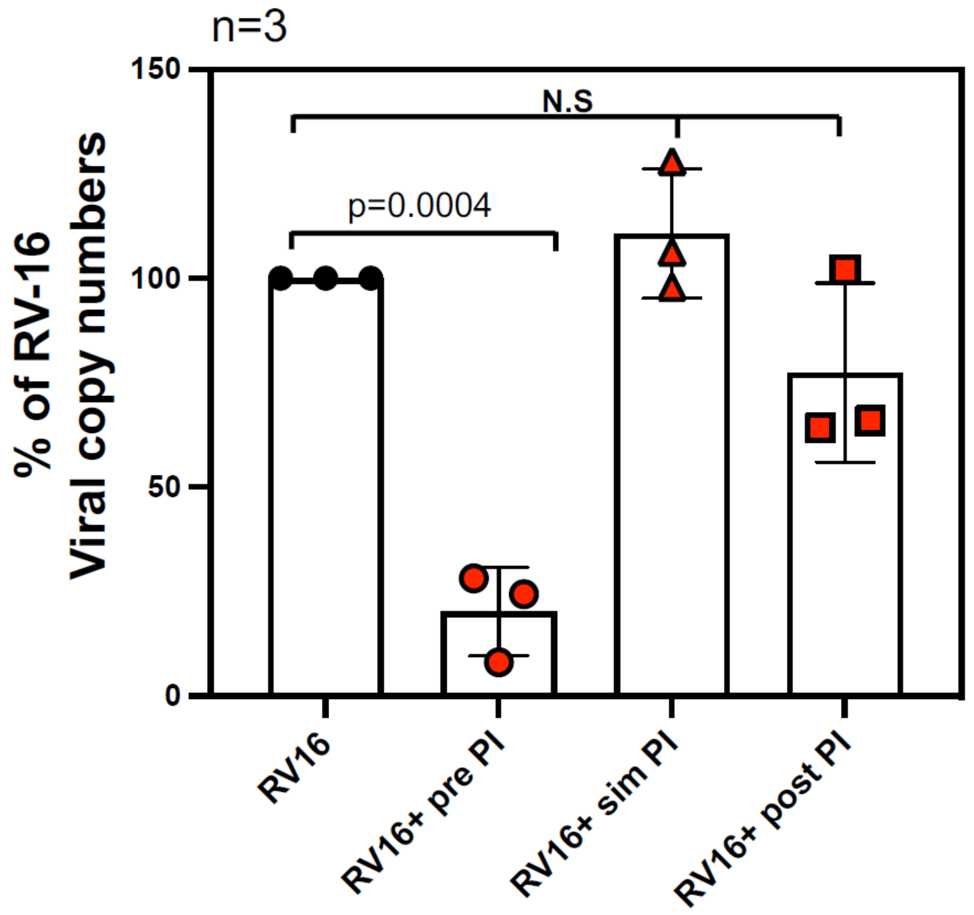

Figure S1. The time course of PI treatment of primary human AECs infected with RV-A16. The AECs from 3 different donors were pretreated with PI for 16 hrs before virus infection (RV16 + pre PI), treated simultaneously with virus infection (RV16 + sim PI), or treated 24 hrs after virus infection (RV16 + post PI). The data are shown as Means ± SD as of RV16 alone as 100% from 3 independent experiments.

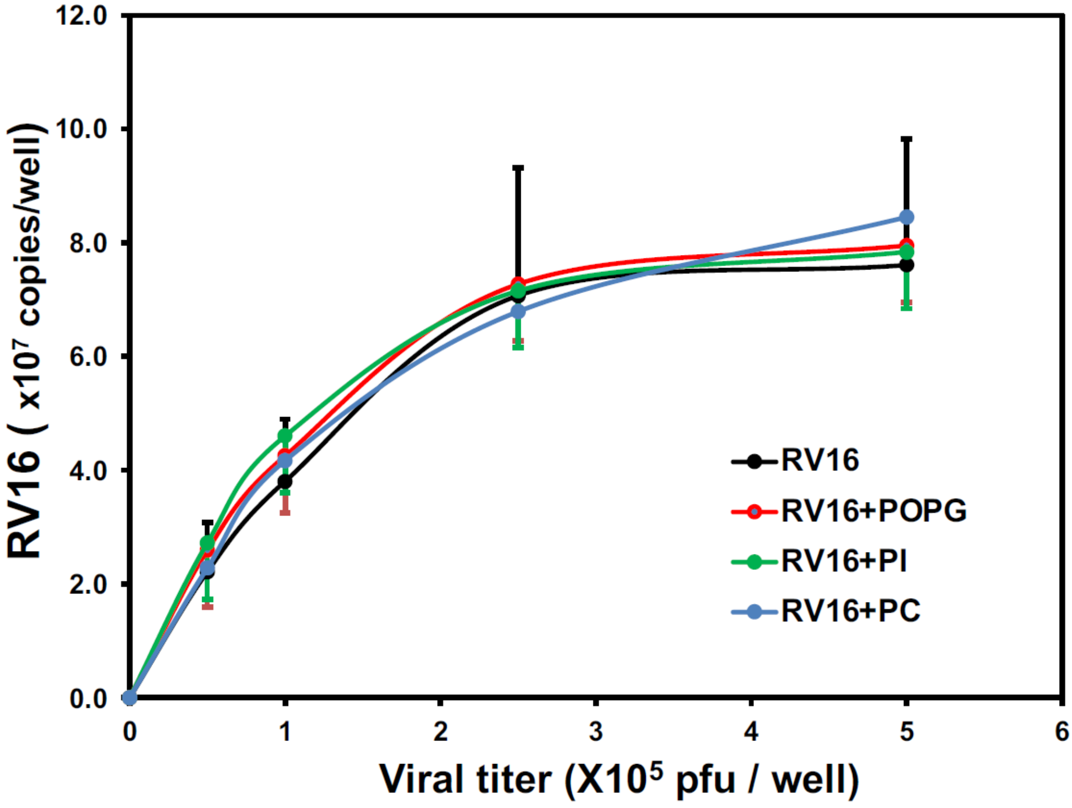

Figure S2. POPG and PI do not inhibit RV-A16 binding to differentiated bronchial epithelial cells and prevent viral internalization in AECs. A) We used 14 day-old differentiated bronchial epithelial cells from 3 different donors. Cells were incubated with RV-A16 at various viral doses (0.5-5 X105 pfu/well) for 4hr at 4 oC (to prevent endocytosis) with or without phospholipids added to the apical sides of the cultures. Cells were washed with DPBS to remove unbound virus and then processed for RNA extractions to measure RV-A16 copy numbers by qRT-PCR. B) We challenged AECs cells in ALI with or without pretreatment of PI (4mg/ml) at 4 oC or 35 oC at various viral doses (1.0, 2.5-5 X105 pfu/well) of RV-A16 for 2hrs. The cells were processed with or without Trypsin/EDTA treatment following RNA extraction to quantitate RV-A16 viral copy numbers in each condition. The data were shown as means ± SD.

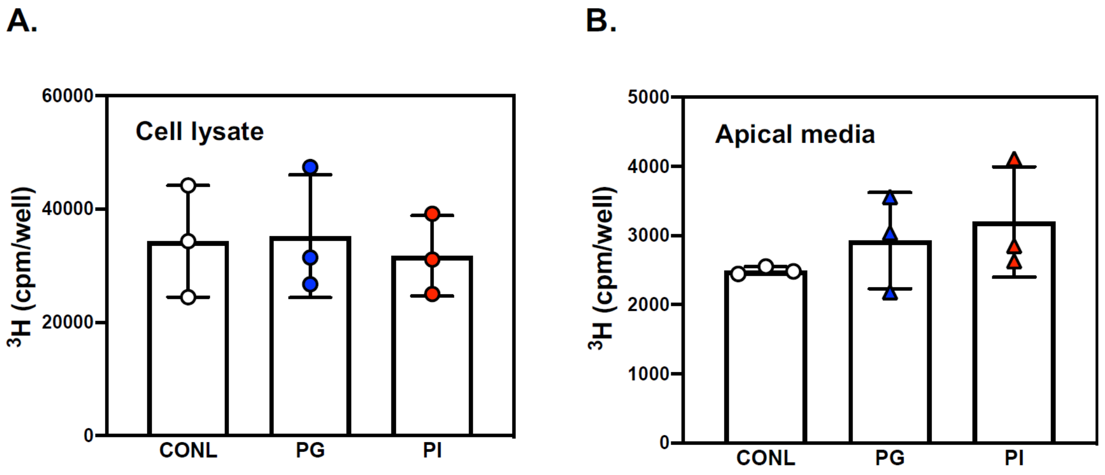

Figure S3. POPG and PI do not inhibit metabolism in primary human AECs. AECs were incubated for 64h with 3H-leucine in the presence or absence of 10 mg/mL of POPG; 4 mg/ml of PI or 20 mg/ml of PC in the apical media. 3H-intracellular protein synthesis (A), and 3H secreted protein in apical media (B) are shown. Values are means ± SD from three independent experiments using 3 different subjects.

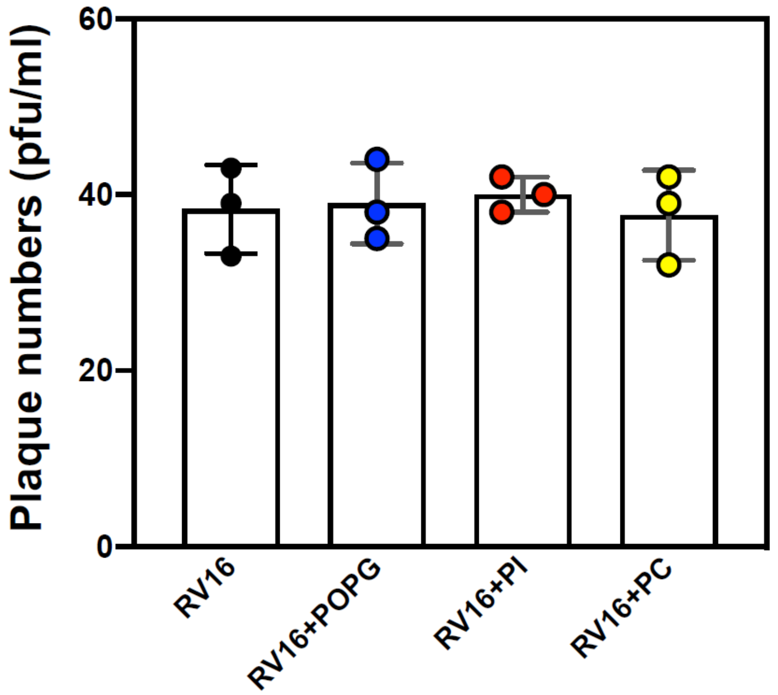

Figure S4. POPG or PI do not have a direct virucidal effect on RV-A16. We incubated RV16 (1X105 pfu) and the lipids (PI 4 mg/ml, POPC 10 mg/ml and PC 20 mg/ml) in 25 μl of AEC culture medium at 35 oC for 4hrs. The mixtures were diluted to 1mL total volume to minimize lipid concentrations and to make series of dilutions (10-3 to 10-5) for RV plaque assay. The numbers are Means ± SD (plaque 5 numbers/well) at a 10-3 dilution (RV16: 38.3± 5.0, RV16 + POPG: 39.0 ± 4.6, RV16 + PI: 40.0 ± 2.0, RV16 + PC 37.7 ± 5.1) from 3 individual experiments.

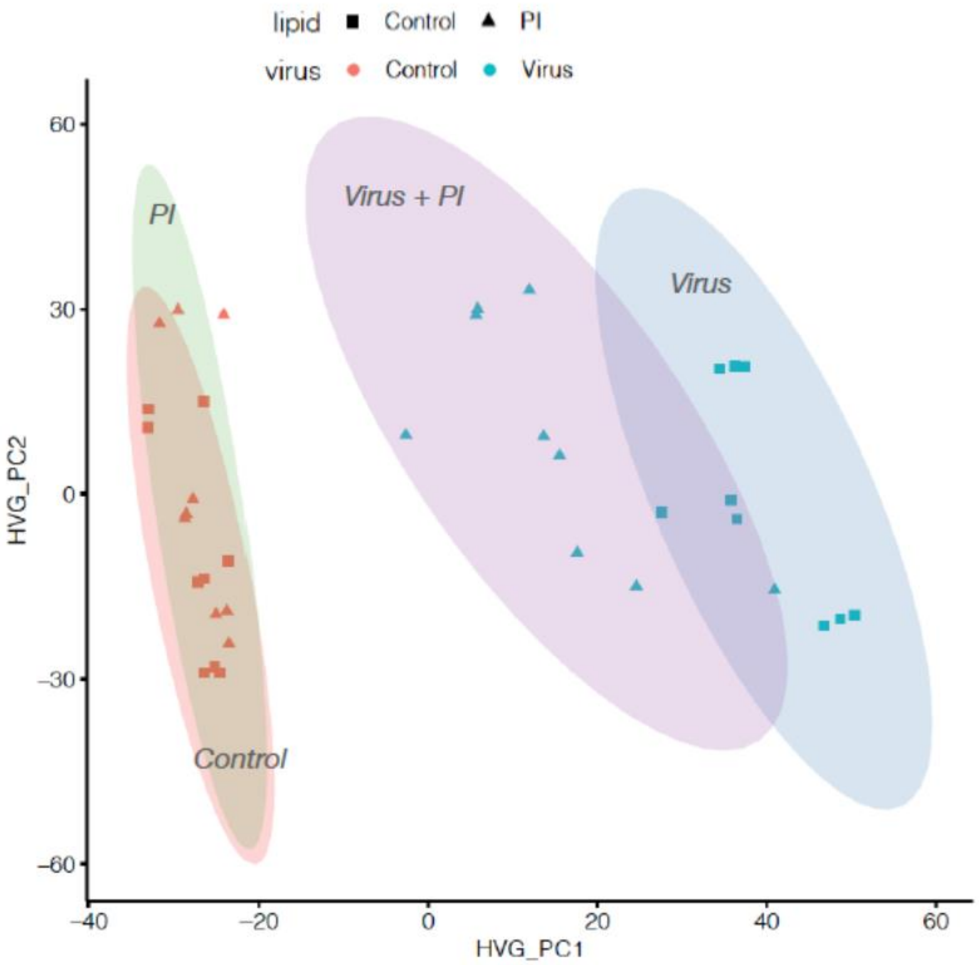

Figure S5. Principal component analysis of mock-infected (Control), RV-A16 (Virus), RV-A16+PI (Virus+PI), and PI samples. Scatterplot of the first two principal components from PCA of the top 10,000 highly variables genes for mockinfected (orange, square), RV-A16 infected (turquoise, square), RV-A16+PI infected/treated (turquoise, triangle), and PI treated (orange, triangle) samples.
